# Does the manual insertion torque of smartpegs affect the outcome of implant stability quotients (ISQ) during resonance frequency analysis (RFA)?

**DOI:** 10.1186/s40729-019-0195-1

**Published:** 2019-12-12

**Authors:** Ingrid Kästel, Giles de Quincey, Jörg Neugebauer, Robert Sader, Peter Gehrke

**Affiliations:** 1 Bad Dürkheim, Germany; 2 Rosmalen, Netherlands; 3 Department of Periodontology, University of Bern, Bern, Switzerland; 4 Interdisciplinary Department of Oral Surgery and Implantology, Department of Craniomaxillofacial and Plastic Surgery, University of Cologne, Cologne, Germany; 5 Landsberg am Lech, Germany; 6 Department for Oral, Cranio-Maxillofacial and Facial Plastic Surgery, Medical Center of the Goethe University, Frankfurt, Germany; 7 Ludwigshafen, Germany; 8 Department of Postgraduate Education, Master of Oral Implantology, Oral and Dental Medicine, Johann Wolfgang Goethe-University, Frankfurt, Germany

**Keywords:** Implant stability quotient (ISQ), Resonance frequency analysis (RFA), Smartpeg, Hand tightening, Insertion torque

## Abstract

**Background:**

There is disagreement about the optimal torque for tightening smartpegs for resonance frequency analysis (RFA). Subjective finger pressure during hand tightening could affect the reliability of the resulting values. The aim of the current study was therefore to assess whether or not the insertion torque of a smartpeg magnetic device influences the implant stability quotient (ISQ) value during RFA.

**Methods:**

Thirty self-tapping screw implants (XiVE S, Dentsply Sirona Implants, Bensheim, Germany) with a diameter of 3.8 mm and a length of 11 mm were inserted in three cow ribs with a bone quality of D1. The RFA value of each implant was measured (Ostell, FA W&H Dentalwerk, Bürmoos, Austria) in two orthogonal directions (mesial and buccal) after tightening the corresponding smartpeg type 45 with a mechanically defined value of 5 Ncm (Meg Torq device, Megagen, Daegu, South Korea) (test). Additionally, 4 different examiners measured the RFA after hand tightening the smartpegs, and the results were compared (control). Insertion torque values were determined by measuring the unscrew torque of hand seated smartpegs (Tohnichi Manufacturing Co. Ltd, Tokyo, Japan).

**Results:**

The ISQ values varied from 2 to 11 Ncm by hand tightening and from 2 to 6 Ncm by machine tightening. The comparison of hand and machine tightening of smartpegs displayed only minor differences in the mean ISQ values with low standard deviations (mesial 79.76 ± 2,11, buccal 77.98 ± 2,) and no statistical difference (mesial *p* = 0,343 and buccal *p* = 0,890).

**Conclusions:**

Manual tightening of smartpeg transducers allows for an objective and reliable determination of ISQ values during RFA.

## Background

While dental implants have become increasingly important in the functional and esthetic rehabilitation of patients, implant failure still does occur. Primary stability at the time of implant placement and the development of osseointegration in the following healing process (secondary stability) are essential parameters for implant success [1]. Primary stability can be considered as the biomechanical stability that holds the implant in place. It is highest immediately after insertion and decreases with time [2]. Primary stability is achieved by the mechanical retention of the implant and is dependent on the design and thread geometry of the fixture. With sufficient implant stability, a shortened healing time and immediate implant function is possible [3]. Primary stability is influenced by the quality and quantity of bone present [4]. In compact cortical bone, it is achieved more frequently than in spongious bone, due to reduced bone density when trabeculae are present [5]. Implant length, diameter, geometry, surface characteristics, insertion technique, and congruence between the drilling site and the implant size [6] are reported as additional influencing factors. Furthermore, the stability of the implant plays a crucial role in the clinical follow-up and control of the degree of osseointegration after implant insertion. Various assessment protocols have been proposed for determining primary implant stability. Subjective methods, such as the surgeon's individual assessment or percussion testing, have little significance, and cannot be reproduced predictably [5, 7]. Objective tests include Periotest® measurements [8–10], the assessment of insertion torque [8, 9, 11], the experimental removal of the implant [12], or a resonance frequency analysis (RFA) [9–11]. RFA was first described by Merideth in 1996 [13]. Modern RFA devices such as the Osstell device (W & H Dentalwerk, Bürmoos GmbH, Austria) can calibrate the raw frequencies for the selected implant system with implant-specific transducers (Smartpegs, W & H Dentalwerk, Bürrmoos GmbH, Austria) and convert them into implant stability quotients (ISQ). According to the manufacturer’s instructions, smartpegs are manually inserted into the implant by means of a plastic insertion aid [14]. The scale of ISQ values ranges from 0 to 100%, with stability increasing with increasing ISQ. The values are not linearly distributed but correspond to a low stability at values below 60, a medium stability at values between 60 and 69, and a high stability at values above 70 [15]. ISQ values at the same implant may clinically differ, depending on which direction is measured. It is therefore recommended that two measurements are carried out from orthogonal directions. It has been controversially discussed whether the individual finger pressure of different examiners may show alterations of ISQ values when hand tightening the smartpegs. A recent in vitro study by Geckili et al. indicated that the manual insertion torque of smartpeg transducer with a plastic driver can have an influence on the results determined [16]. Consequently, the authors recommended the manufacturer to standardize the tightening of smartpegs to a range of 5–8 Ncm to obtain reliable and objective RFA values, instead of leaving it to subjective finger pressure. As there is only limited in vivo or ex vivo data available to confirm or refute these results, the aim of the current study was to assess whether or not the insertion torque of a smartpeg transducer influences the ISQ values identified. The null hypothesis was that the manual tightening force of a smartpeg device has an impact on the resulting ISQ values during resonance frequency analysis.

## Material and methods

**Fig. 1:**
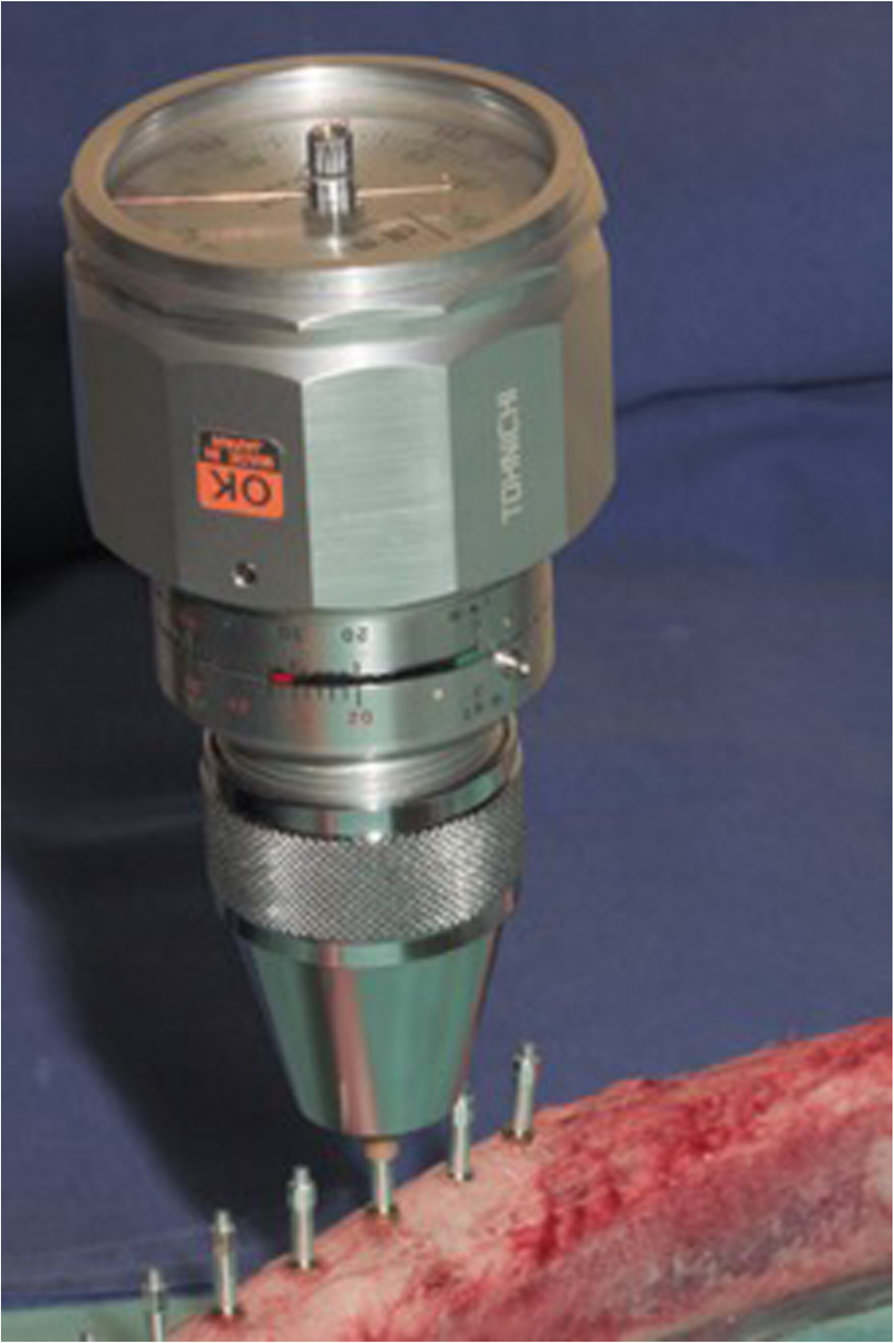
Insertion torque and unscrewing device. Determination of insertion torque of individually hand-tightened smartpegs in test group by recording the removal torque required when unscrewing the device (BTG36N Analog Torque Meter, Tohnichi Manufacturing Co. Ltd., Tokyo, Japan)

**Fig. 2:**
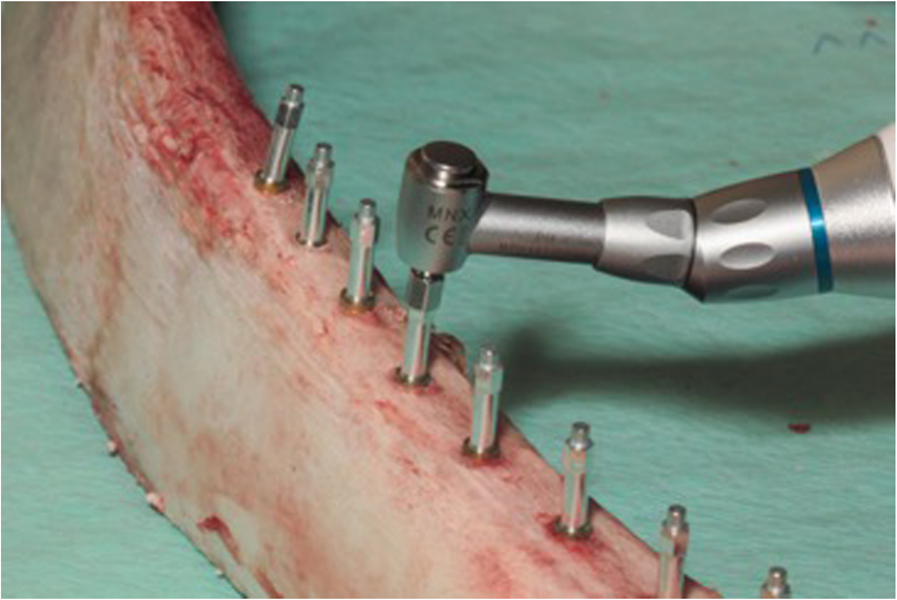
Smartpeg on implants. Mechanical insertion of smartpegs into implants in control group with a defined insertion torque of 5 Ncm using an electronical Meg Torq device (Megagen Implants UK, Luton Bedfordshire, UK)

Three fresh bovine ribs from the same animal were selected for the current in vitro testing; the bovine ribs were of a similar size to those used by Gecikli et al. [16], thus attempting to imitate human edentulous bone with a similar composition of cortical and cancellous bone. The animal was farmed and sacrificed for food production. The bone was stored airtight, humid, and cool from the time the cow was dissected until the study was carried out. The implant sites were prepared following the standard protocol recommended by the manufacturer, and 30 self-tapping screw implants (XiVE S, Dentsply Sirona Implants, Bensheim, Germany) with a diameter of 3.8 mm and a length of 11 mm were inserted into the ribs with a bone quality of D1 (10 each) with a safe distance to each other. Since bone quality and surgical technique have an influence on the collected ISQ values [17, 18], Implant placement was performed by the same surgeon (IK). According to the manufacturers’ recommendation, the bone was center marked with a round bur at 800 rpm. This was followed by a pilot drill, an enlarging drill, and the final drill of D 3.8 mm. Because of high bone density (D1) preparation of the osteotomy was followed by a crestal countersink preparation at 15 rpm. All implants were inserted at 50 Ncm and the insertion abutments (XiVE TempBase, Dentsply Sirona Implants, Bensheim, Germany) were removed. In a test group, four different surgeons (S1–S4) with different skill levels and with different backgrounds of experience of RFA hand tightened the corresponding smartpeg components (Type 45, Ostell, FA W & H Dentalwerk, Bürmoos, Austria) into all implants. All examiners were blinded to the study protocol. Subsequently, ISQ values of the 30 implants were measured by each examiner (S1–S4) utilizing RFA (Ostell, IDx, FA W & H Dentalwerk, Bürmoos, Austria) from two orthogonal directions (mesial/buccal). The probe of the analyzer was seized 1 mm from the smartpeg transducer at a 90° angle, and the RFA value was registered as implant stability quotient (ISQ). To determine the insertion torque of the individually hand-tightened smartpeg by each examiner, the removal torque required when removing the device was recorded. This was carried out by using a BTG36N Analog Torque Meter (Fig. 1) (Tohnichi Manufacturing Co. Ltd., Tokyo, Japan). In a control group, the appropriate smartpeg magnetic devices were mechanically inserted into all implants using an electronical Meg Torq device (Fig. 2) (Megagen Implants UK, Luton Bedfordshire, UK) with a defined insertion torque of 5 Ncm. Prior to each insertion, the Meg Torq device was calibrated according to the manufacturer's specifications. Again, the ISQ values of all implants were measured from two orthogonal directions. To verify the insertion torque of the mechanically tightened smartpegs, the removal torque required to unscrew the transducer was recorded (Tohnichi Manufacturing Co. Ltd, Tokyo, Japan).

Statistical analysis was performed to evaluate the difference between the test and control group. Variance analysis and continuous variables were determined. Pearson *r* correlation coefficient was tested to verify the relationship between the two variables (e.g., relationship between torque and ISQ). Statistical significance was set at *p* > 0.05.

## Results

**Fig. 3:**
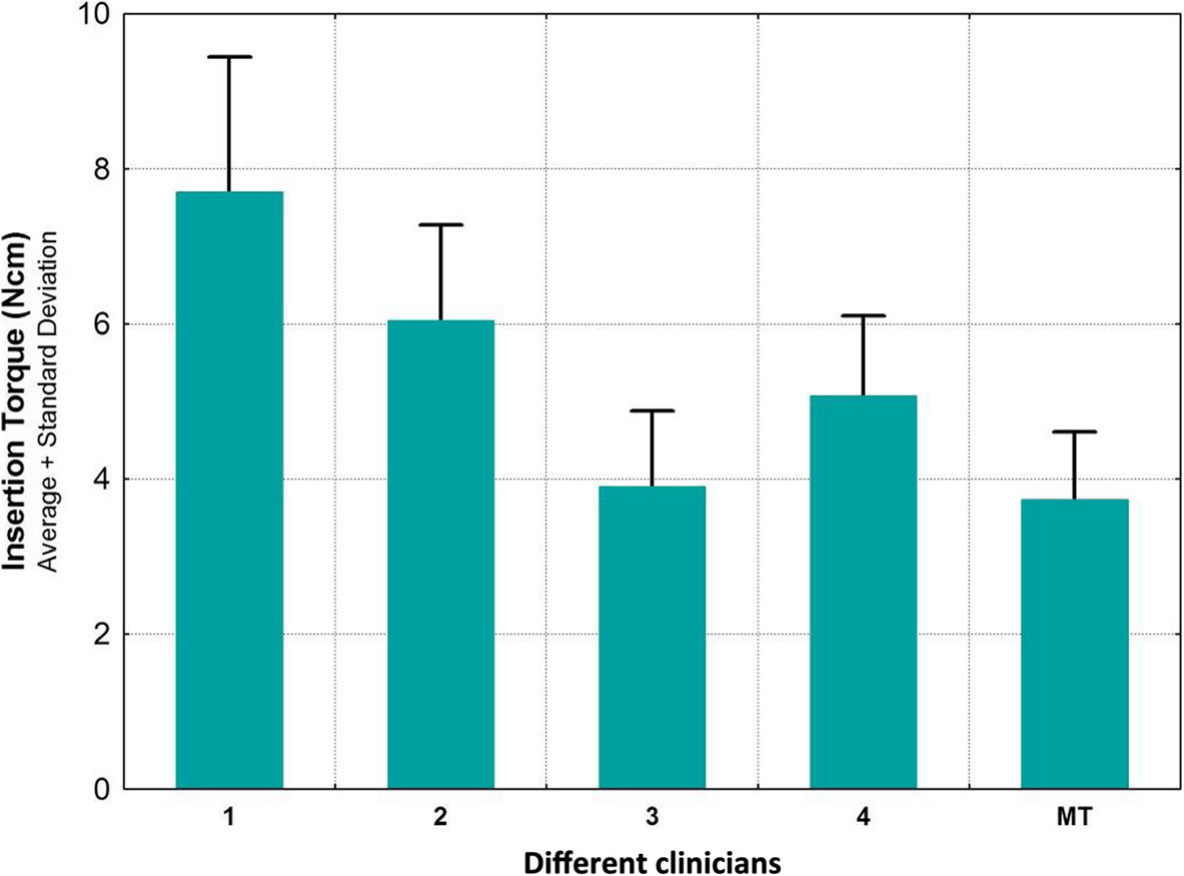
Frequency distribution among test group (hand-tightened SmartPegs by examiners 1 to 4) and control group (MT = mechanically inserted SmartPegs) according to insertion torque

**Fig. 4:**
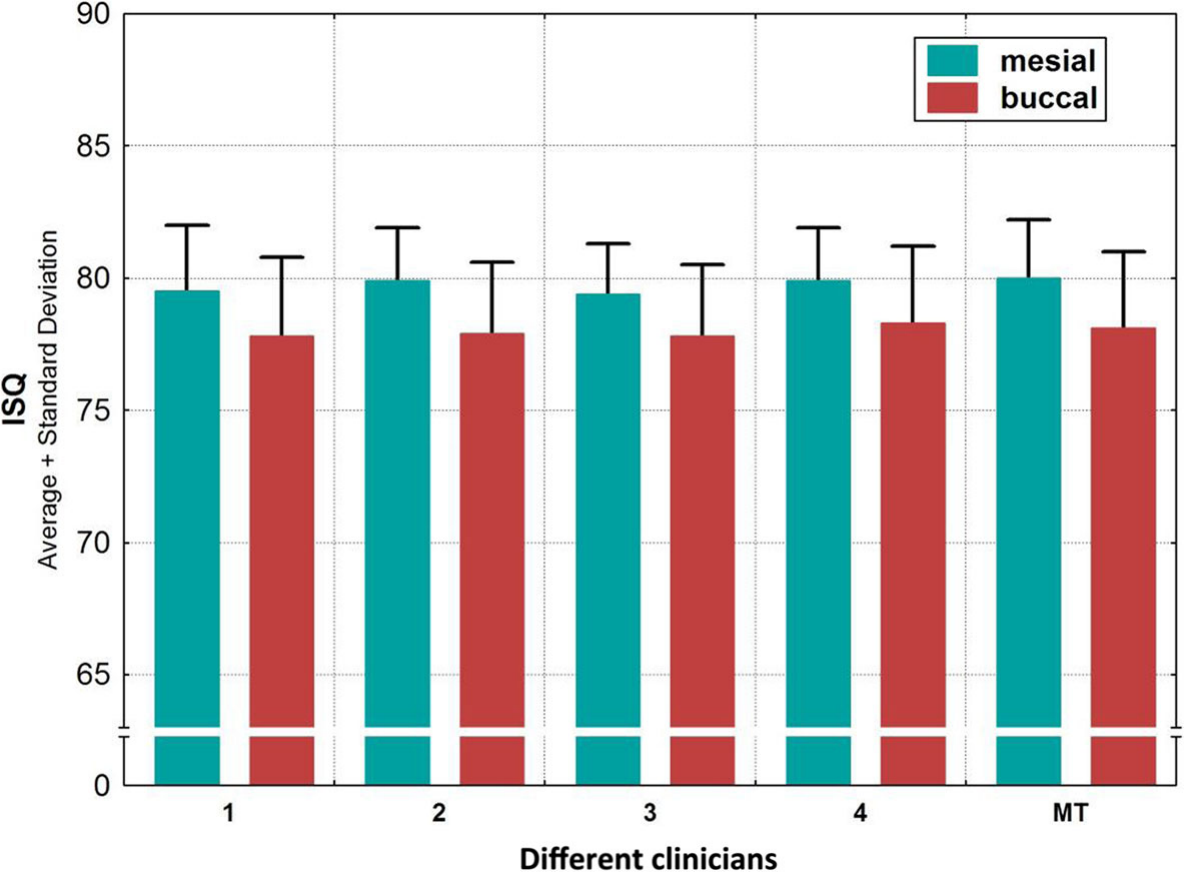
Frequency distribution among test group (hand-tightened SmartPegs by examiners 1 to 4) and control group (MT = mechanically inserted SmartPegs) according to ISQ values

In the test group, mesial and buccal ISQ values of 30 implants were recorded by four examiners (S1–S4) after hand tightening the smartpegs. The control group considered mesial and buccal ISQ values of 30 implants with mechanically inserted smartpegs. Only 147 values could be recorded because one transducer broke and thereafter no further values could be obtained for this implant. At torque values between 2 and 11 Ncm (mean 5.3, median 5), ISQ values between 71 and 85 could be registered. All values were correlated to a high primary stability (ISQ > 70). Table 1 displays the ranked variables ISQ mesial, ISQ buccal and torque computed. At torques between 2 and 11 Ncm (Fig. 3), the mean values of the ISQ values were mesially between 79.1 and 80.8 and buccally between 74.5 and 79.0 (Fig. 4). The buccally measured values were generally lower. A possible explanation for this finding could have been the existence of a thinner buccal bone wall of the utilized bovine ribs. A comparison of all measured values (*n* = 147) including the manual torque achieved by the four examiners and the mechanical torque achieved by the Meg-Torq device to seat a smartpeg displayed only a limited influence on the outcome of the resulting ISQ values (Table 2). Results for the calculation of the ratio between insertion torque and ISQ values were recorded and differences between the subjects were computationally eliminated. In this case, no statistically significant influence of the torque used to place a smartpeg transducer on the registered ISQ value could be demonstrated, neither for mesial (*p* = 0.343) nor for buccal (*p* = 0.890) ISQ values (Table 3).

**Table 1:** Parameters for continuous variables: ISQ mesial, ISQ buccal and insertion torque.

Descriptive statistics						
	Validity *N*	Average	Median	Minimum	Maximum	Stand. deviat.
ISQ mesial	147	79.76190	80.00000	72.00000	84.00000	2.111071
ISQ buccal	147	77.97959	78.00000	71.00000	85.00000	2.836816
Insertion torque (Ncm)	147	5.30612	5.00000	2.00000	11.00000	1.907492

**Table 2:**
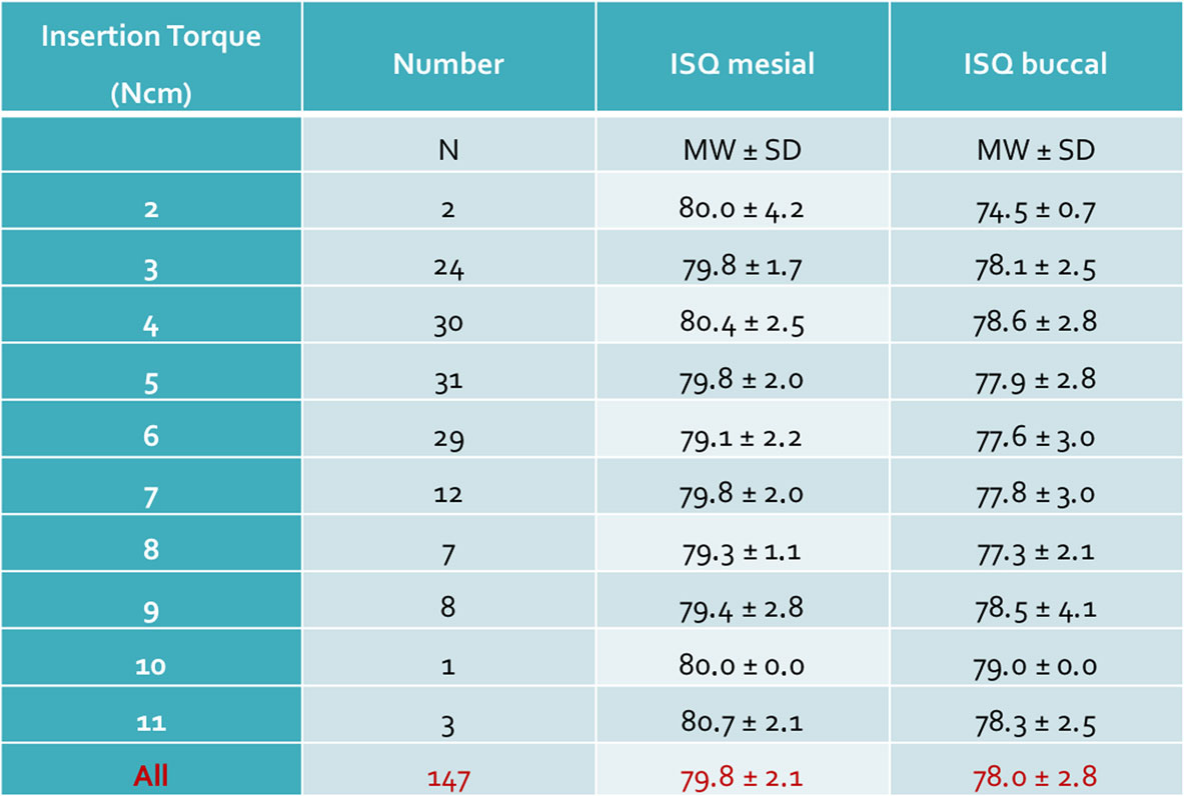
Insertion torque relative to ISQ value for all implants

**Table 3:** The correlation of insertion torque to ISQ showed no statistical significance (italics)

Correlation	*r* (*X*,*Y*)	*r*^2^	*t*	*p*	*N*
Torque (Ncm) ISQ mesial					
	− 0.078728	0.006198	− 0.950961	*0.343207*	147
Torque (Ncm) ISQ buccal					
	− 0.011495	0.000132	− 0.138430	*0.890093*	147

## Discussion

A great number of studies have summarized that the measurement of implant stability with RFA is reliable, is noninvasive, and can be used at any time after implant insertion and during follow-up [19, 20]. Various parameters have been demonstrated to influence the degree of primary implant stability [21, 22]. These include bone density [23, 24], surgical technique [25], implant insertion torque [26], and congruence between the osteotomy and implant diameter [27]. Recently, it has been suggested that RFA measurements made by magnetic devices may show variable results of implant stability due to different forces applied during transducer tightening by different clinicians [20]. Although widely used in implant therapy and research, it has been critically discussed whether or not a manual connection of the transducer device may have a negative impact on the accuracy of ISQ measurements, thus introducing an important element of bias on an individual operator basis [28]. Meredith et al. were one of the first in 1996 to describe a correlation between the insertion torque of a transducer in the first generation of Ostell devices [13]. They reported that despite of a good reproducibility of ISQ values, different insertion torques of the transducers may change the ISQ values. According to Herrero-Climent et al., this no longer applies to today's devices [29]. The manufacturer proposes that smartpegs should be tightened to 4–5 N, which is described as finger tight. However, finger tightness is variable and is not an objective criterion. Geckili et al. conducted an in vitro investigation to determine the optimal value for tightening smartpeg devices [16]. The study set-up of the present in vitro trial was designed in close accordance with the trial of Geckili et al. in order to facilitate a direct comparison to the results obtained. Since macro and micro design of an implant can influence its primary stability, 30 self-tapping screw implants (XiVE S, Dentsply Sirona Implants, Bensheim, Germany) with identical surface texture, length and diameter were placed by the same experienced implant surgeon. All implants were placed into comparably thick and dense bovine ribs (D1 bone). Thus, minimizing the undesired impact of the before mentioned factors on the study outcome. While the implants inserted in the present study offered an internal hexagon as implant-abutment connection for the seating of the transducers, the reference investigation of Geckili et al. utilized implants with an internal octagon-tube connection (Trias implant system, Servo-Dental GmbH & Co. KG, Hagen, Germany). Because of this similar internal implant-abutment connection, it may be expected that the results of both in vitro studies are comparable, since they are based on an equivalent functionality. Individual smartpeg devices are available for each major implant system. According to the manufacturer, the ISQ values of various implant systems may vary up to 5 value steps, but not within one system. Since only one implant system was used in each of both aforementioned studies, it can be regarded as irrelevant in the respective evaluation. Since macro and micro design of an implant can influence its primary stability, the influence of the internal geometry of implants with different implant-abutment connections has not been investigated. We should not transfer these results to other types of implants due differences to other implant-abutment connections. In order to represent clinicians at different skill levels, two female and two male subjects were selected as examiners with different backgrounds of experience with respect to implant dentistry. As expected, the respective examiners hand tightened the smartpegs with different torque values. The lowest torque value achieved was 2 Ncm. When considering an insertion force of 1 Ncm, as tested in the in vitro trial of Gickeli et al., the transducer device is inserted so minimally, that it is mostly just seated into the implant. None of the clinicians in the current study achieved such a low level of force during manual insertion. This low torque value is more likely to be based on a hypothetical assumption or a constructed measurement, which seems to be clinically irrelevant. In the majority of cases, the manual torque achieved by the examiners in our test group ranged between a value of 3 and 6 Ncm. The maximum manually achieved value was 11 Ncm. In one case, the smartpeg fractured when seating it into the implant. Subsequently, no further measurement was possible at this implant. This supports the manufacturer's recommendation that smartpeg devices are for single use only and should, for safety reasons, not be reused. In the control group, the transducers were mechanically inserted with a predefined torque of 5 Ncm. This was in accordance with the study results of Geckili et al. who recommended a defined insertion torque value of 5 to 8 Ncm to install a magnetic device for RFA. We decided for a predefined torque value of 5 Ncm as being the preferred value to be investigated, to ascertain whether there is a difference between the measured ISQ values at the preset 5 Ncm and the manually achieved 5 Ncm values. A difference in this aspect could however not be demonstrated (Fig. 3). It should be noted that the torque to insert a smartpeg transducer does not necessarily correspond to its removal torque during unscrewing. In accordance to the literature [30–32], the removal torque values of the mechanically seated smartpegs in the present study were continuously below 5 Ncm with a range of 79–93% of the initial insertion torque. With increasing torque, the ISQ values tended to decrease slightly. There was a decrease of 0.09 for the ISQ at the mesial and 0.02 for the ISQ at the buccal aspect per Ncm increase in torque. Within the present range of torque values (2 to 11), the mean ISQ values decreased by 0.81 (mesial) and 0.18 (buccal) points, respectively. Accordingly, no dependency of insertion torque of the smartpeg transducer and the corresponding ISQ value could be observed (Table 3). Regardless of the individual examiner and the torque applied to seat the smartpeg, comparable ISQ values were attained during RFA. A manual insertion seems to be sufficient, regardless of the skill level of the clinician.

## Conclusions

The null hypothesis that finger tightening of transducer devices for RFA analysis may have a negative impact on the accuracy of ISQ measurements was rejected. Different forces applied during transducer tightening by different clinicians had no significant effect on the resulting ISQ values. Manual tightening of smartpeg magnetic devices allows for an objective and reliable determination of ISQ values during RFA.

## Data Availability

The datasets used and analyzed during the current study are available from the corresponding author on reasonable request.

## Abbreviations

RFA: Resonance frequency analysis
ISQ: Implant stability quotient
